# The Histone H3 Lysine 27-Specific Demethylase Jmjd3 Is Required for Neural Commitment

**DOI:** 10.1371/journal.pone.0003034

**Published:** 2008-08-21

**Authors:** Thomas Burgold, Fabio Spreafico, Francesca De Santa, Maria Grazia Totaro, Elena Prosperini, Gioacchino Natoli, Giuseppe Testa

**Affiliations:** European Institute of Oncology, Milan, Italy; Duke University, United States of America

## Abstract

Patterns of methylation at lysine 4 and 27 of histone H3 have been associated with states of gene activation and repression that are developmentally regulated and are thought to underlie the establishment of lineage specific gene expression programs. Recent studies have provided fundamental insight into the problem of lineage specification by comparing global changes in chromatin and transcription between ES and neural stem (NS) cells, points respectively of departure and arrival for neural commitment. With these maps of the differentiated state in place, a central task is now to unravel the chromatin dynamics that enables these differentiation transitions. In particular, the observation that lineage-specific genes repressed in ES cells by Polycomb-mediated H3-K27 trimethylation (H3-K27me3) are demethylated and derepressed in differentiated cells posited the existence of a specific H3-K27 demethylase.

In order to gain insight into the epigenetic transitions that enable lineage specification, we investigated the early stages of neural commitment using as model system the monolayer differentiation of mouse ES cells into neural stem (NS) cells. Starting from a comprehensive profiling of JmjC-domain genes, we report here that Jmjd3, recently identified as a H3-K27me3 specific demethylase, controls the expression of key regulators and markers of neurogenesis and is required for commitment to the neural lineage.

Our results demonstrate the relevance of an enzymatic activity that antagonizes Polycomb regulation and highlight different modalities through which the dynamics of H3-K27me3 is related to transcriptional output. By showing that the H3-K27 demethylase Jmjd3 is required for commitment to the neural lineage and that it resolves the bivalent domain at the Nestin promoter, our work confirms the functional relevance of bivalent domain resolution that had been posited on the basis of the genome-wide correlation between their controlled resolution and differentiation. In addition, our data indicate that the regulation of H3-K27me3 is highly gene- and context- specific, suggesting that the interplay of methyltransferases and demethylases enables the fine-tuning more than the on/off alternation of methylation states.

## Introduction

On the basis of biochemical evidence indicating that the turnover of histone methyl marks was equivalent to the turnover of histones themselves [1], the prevailing view held that these marks were stable and that histone lysine methylation (HLM) could be reversed only by histone replacement. In turn, this stability led to posit a central role for HLM in the establishment and maintenance of lineage specific gene expression. While challenging the inherent stability of the methylation mark, the discovery of histone lysine demethylases (HDMs) is consistent with the observation that HLM at specific loci is responsive to environmental cues and is regulated during differentiation [2], [3], [4]. In fact, the two sets of observations–the apparent lack of global methylated histone turnover and the existence of specific HDMs– need not be in conflict. It is precisely the combination of a very regulated process of addition and removal of methyl marks with the stability of these marks thereafter that makes it reasonable to hypothesize for HLM a pivotal role in lineage commitment. In this context, the realization that Ezh2, a member of the Polycomb group (PcG) of proteins discovered in *Drosophila* as stable repressors of the *Hox* cluster, catalyzes H3-K27 methylation suggested a central role for this modification in the process of gene silencing that accompanies differentiation. Indeed H3-K27 methylation and binding of PcG proteins are dynamically regulated during differentiation [5], [6], [7], [8] and two main patterns have emerged from recent genome-wide studies in *Drosophila* and mammals [9]. In both ES cells and neural progenitors several genes bound by PcG proteins and marked by H3-K27me3 are repressed and become activated during differentiation. As many of these genes are key developmental regulators, the current model holds that PcG protein-mediated repression prevents inappropriate differentiation. Other genes however, comprising up to 20% of PcG protein targets, appear to be actively expressed despite PcG protein binding and H3-K27me3 [5], [6]. This indicates that, as with many other posttranslational modifications of histones, also the H3-K27me3 mark needs to be ‘read’ and ‘translated’ into the appropriate functional output and posits a more complex and nuanced set of functions for PcG proteins [9].

Furthermore, several loci in both ES and adult stem cells, including those encoding key developmental regulators, are characterized by the simultaneous presence of H3-K4me3 and H3-K27me3, a configuration described as “bivalent domain” [3], [10]. In undifferentiated ES cells this unusual combination of marks is thought to keep genes repressed or expressed at very low levels but poised for later activation. At the end of the transition from ES to neural stem cells (NS), a large fraction of these bivalent domains appears to have been resolved in a lineage specific fashion leaving univocal signatures of either activation (H3-K4me3) or repression (H3-K27me3).

In order to explore the relevance of histone demethylation to lineage commitment, we profiled the expression of JmjC-domain HDMs during ES cell differentiation and found that Jmjd3, a recently recognized H3-K27 demethylase, is required for the commitment of ES cells to the neural lineage. We report here that Jmjd3 directly controls key regulators and markers of neurogenesis, and we highlight different modalities through which the dynamics of H3-K27 trimethylation is related to transcriptional output.

## Results

**Figure 1:**
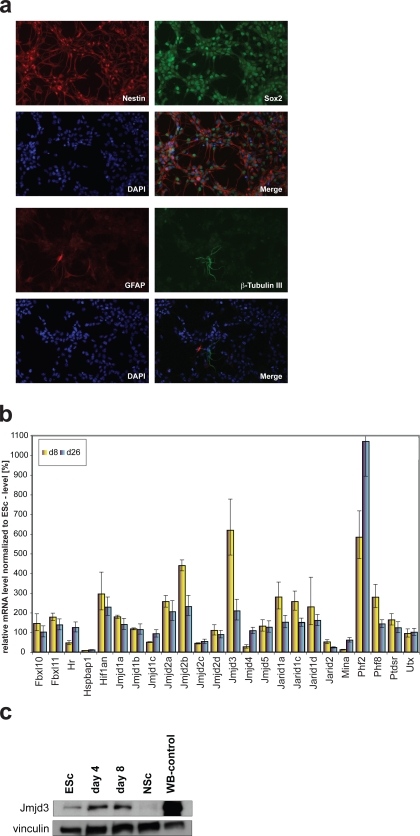
Expression profiling of JmjC-domain containing proteins identifies Jmjd3 as a protein specifically upregulated at the outset of neural commitment. (a) ES cell-derived neural stem (NS) cells constitute a homogeneous population of neural precursors. They show uniform expression of neural precursor markers nestin and Sox2 and virtually no immunoreactivity for neuronal (β-tubulin III) or astrocyte (GFAP) antigens. (b) Expression of JmjC-genes at day 8 and 26 of ES cell to NS cell differentiation. mRNA levels were quantified by real-time RT-PCR. For each gene the transcript level in wild-type ES cells was set as 100%. The bars represent the means±S.D. of triplicates normalized to TBP. (c) Protein expression levels of Jmjd3 during the ES cell to NS cell transition. Levels of Jmjd3 peak at days 4 and 8 and are downregulated in established NS cell cultures. Protein extract from cells overexpressing Jmjd3 was used to localize the Jmjd3 band (WB-western blot control). Vinculin served as a loading control.

We profiled the expression of JmjC-domain HDMs during the differentiation of ES cells into NS cells in adherent monocultures [11]. By avoiding the heterogeneity associated with standard protocols of ES cell differentiation, this experimental system allowed us to specifically trace the relevance of HDMs for the commitment to the neural lineage. We generated homogeneous cultures of NS cells that express immature neural precursor markers (like Sox-2 and Nestin) and lack markers of terminal neuronal (Beta-III tubulin) or glial (Gfap) differentiation (fig. 1a). Expression of JmjC-domain HMDs was analyzed by quantitative RT-PCR (qRT-PCR) at day 8 and day 26 of NS cell derivation (fig. 1b). At day 8 of the differentiation protocol, neural precursors were replated in the presence of EGF and FGF-2, progressively enriching the culture for bipolar cells that were passaged regularly until a homogeneous culture of NS cells was obtained (corresponding to our day 26 sample). Individual JmjC genes showed distinct patterns of expression: several genes were expressed at stable levels, whereas others increased or decreased progressively during the course of differentiation. On the contrary, five JmjC genes (Jmjd2b, Jmjd3, Jarid1a, Jarid1c and Phf8) were first up-regulated at day 8 (differentiating neural precursors) and then down-regulated at day 26 (established and self-renewing neural stem cells), suggesting that they could be involved in the onset of neural commitment. Among these JmjC genes, Jmjd3 showed the greatest up-regulation at day 8 (6 fold increase) with a decrease to near basal levels at day 26; hence we focused on the functional dissection of its role in the early stages of ES cell commitment. We raised a polyclonal antibody against the C-terminus of Jmjd3 and found that the protein peaked already at day 4 of neural differentiation (fig. 1c).

Jmjd3 belongs to the subgroup of JmjC-domain proteins that includes Utx and Uty [12]. We and others recently demonstrated that Jmjd3 and Utx are H3-K27-specific demethylases (see Supplementary figures S1, S2 and S3 and [13], [14], [15], [16]). Thus, the observation that a H3-K27 demethylase is specifically upregulated at the start of ES cell differentiation suggested that its activity may be causally involved in the dynamic regulation of the H3-K27me3 mark that accompanies differentiation.

### Jmjd3 is required for neural commitment

**Figure 2:**
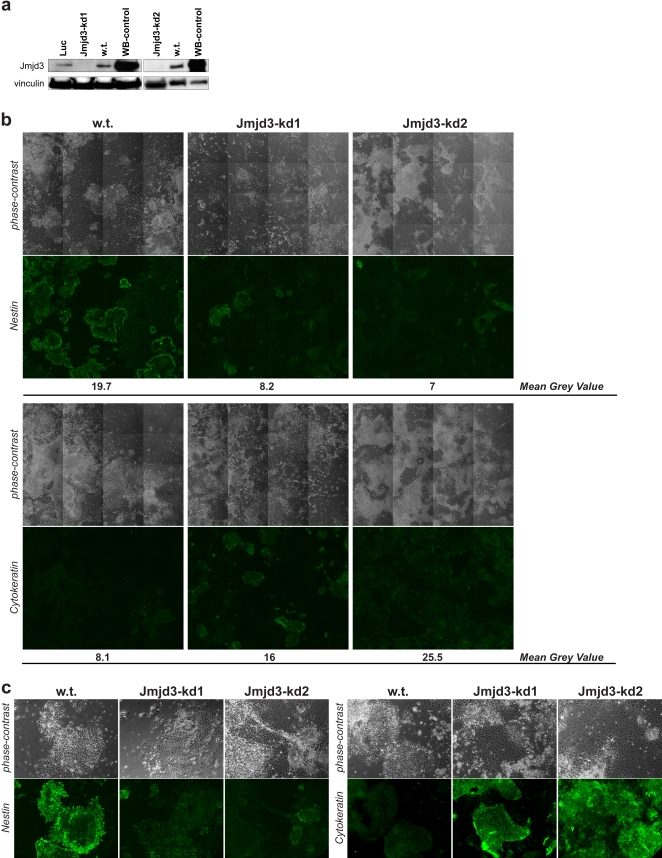
Jmjd3 is required for neural commitment. (a) Protein levels of Jmjd3 in RNAi knock-down ES cell clones assessed by Western Blot. Vinculin served as a loading control. (b) Immunostaining for nestin (upper panel, second row) and pan-cytokeratins (lower panel, second row) and phase-contrast images (first rows of both panels) of wild type (w.t.) and Jmjd3 knock-down (Jmjd3-kd1 and Jmjd3-kd2) cells at day 7 of monolayer differentiation. Sixteen contiguous squares, providing representative coverage of the culture dishes, were imaged at 10X magnification with the same exposure time for wild type and Jmjd3 knock-down cells. Mean grey values were calculated with the ImageJ software. (c) Close-ups showing illustrative examples of the differentiating cultures shown in (b) Immunostaining for nestin (left panel, second row) and pan-cytokeratins (right panel, second row) and phase-contrast images (first rows of both panels) of wild type (w.t.) and Jmjd3 knock-down (Jmjd3-kd1 and Jmjd3-kd2) cells at day 7 of monolayer differentiation imaged with the same exposure time for wild type and Jmjd3 knock-down cells.

We therefore tested the functional relevance of Jmjd3 by RNAi-mediated gene knock-down. We established stable ES cell clones infected with lentiviruses or retroviruses expressing short hairpin RNAs against Jmjd3 under the control of the U6 RNA Pol III-dependent promoter or the MSCV LTR RNA Pol II-dependent promoter, respectively. We used two short hairpin RNAs (shRNAs) targeting different regions of the Jmjd3 mRNA as well as a control hairpin targeting luciferase. For each shRNA we isolated several ES cell clones and characterized them for Jmjd3 expression levels by qRT-PCR. We selected for further characterization one clone expressing the Jmjd3-1 shRNA (clone Jmjd3-kd 1), one clone expressing the Jmjd3-2 shRNA (clone Jmjd3-kd 2) and one control clone expressing the luciferase RNAi hairpin (clone Luc). qRT-PCR showed for clone Jmjd3-kd 1 more than 90% reduction in Jmjd3 mRNA and slightly higher Jmjd3 residual levels in clone Jmjd3-kd 2. Whereas levels of Jmjd3 in the control clone expressing the luciferase RNAi hairpin were not affected, both Jmjd3-kd clones showed markedly reduced Jmjd3 protein levels (fig. 2a), thus indicating that Jmjd3 is not required for ES cell survival. Levels of the pluripotency markers Oct4 and Nanog were also not affected by Jmjd3 depletion (Supplementary fig. S4).

**Figure 3:**
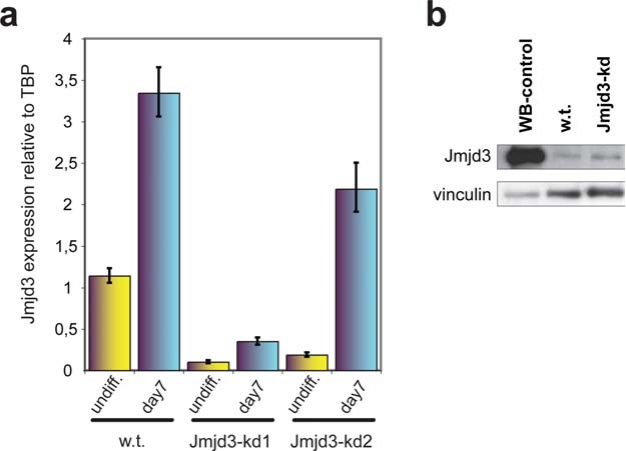
Jmjd3 levels during differentiation. (a) qRT-PCR analysis of Jmjd3 levels in wild type and Jmjd3-kd clones during differentiation. Bars represent the means±s.d. of triplicates normalized to TBP. (b) Levels of Jmjd3 protein in NS cells derived from Jmjd3-kd and control ES cell clones (w.t.) were assessed by Western Blot analysis. Vinculin served as a loading control.

We then sought to differentiate Jmjd3 knock-down (Jmjd3-kd) cells into NS cells and carried out a morphological and lineage marker analysis. Whereas wild type cells (fig. 2b, upper panel and 2c, left panel) and cells expressing the control luciferase RNAi hairpin (Supplementary fig. S5, left panel) formed neurulating rosettes which developed into dense clusters with intricate outgrowths of bi- or tri-polar precursors, Jmjd3-kd ES cells formed these structures at much lower frequency, with the majority of cells flattening out and acquiring a polygonal shape. Cultures of wild type control cells and cells expressing the control luciferase RNAi hairpin showed the typical pattern of neurulating clusters from which tightly juxtaposed neural precursors that are intensely Nestin-positive protrude with characteristic palisade-like shapes (fig. 2b, upper panel and 2c, left panel, showing close-ups of illustrative examples and Supplementary fig. S5, left panel). Both Jmjd3-kd clones had a markedly different phenotype, with much fewer Nestin positive clusters (in which signal intensity was anyway lower than in control cells). Moreover, the signal pattern of these Nestin clusters appeared diffuse and less structured, with only sporadic cases of radial growth of tightly packed precursors. Instead these abnormal clusters were surrounded by extensive sheets of flat cytokeratin positive cells which comprised the majority of cells in Jmjd3-kd cultures, whereas they were observed only in sporadic patches in the wild type control cultures (fig 2b, lower panel and 2c, right panel, showing close-ups of illustrative examples) and in the control cultures expressing the luciferase RNAi hairpin (Supplementary fig. S5, right panel), as expected from the monolayer differentiation protocol. We then asked whether these Jmjd3-kd Nestin clusters would proceed in neurulation and found that they eventually managed to generate NS cells. Because the process of NS cell derivation entails a strong selection that progressively enriches for neural precursors we hypothesized that the few Jmjd3-kd ES cells that eventually managed to neurulate may have been those with residual levels of Jmjd3 that would enable them to initiate neural differentiation. Consistently with this prediction at day 7 of neural differentiation, Jmjd3-kd cells with both hairpins showed a significant upregulation of Jmjd3 (fig. 3a), with higher levels in clone Jmjd3-kd 2 as expected on the basis of the lower knock-down efficiency already observed for this hairpin in the undifferentiated state. As the RNAi machinery is active during differentiation, the upregulation of Jmjd3 in knock-down clones at day 7 reflects most likely the selection pressure that enriches the culture for those cells with less efficient knock-down and higher levels of Jmjd3. In agreement with this model, the NS cells derived from the few Jmjd3-kd ES cells that initiated neurulation have indeed reacquired normal Jmjd3 protein levels (fig. 3b), suggesting that the ES to NS cell transition depends on the presence of Jmjd3.

### Jmjd3 regulates neural markers

**Figure 4:**
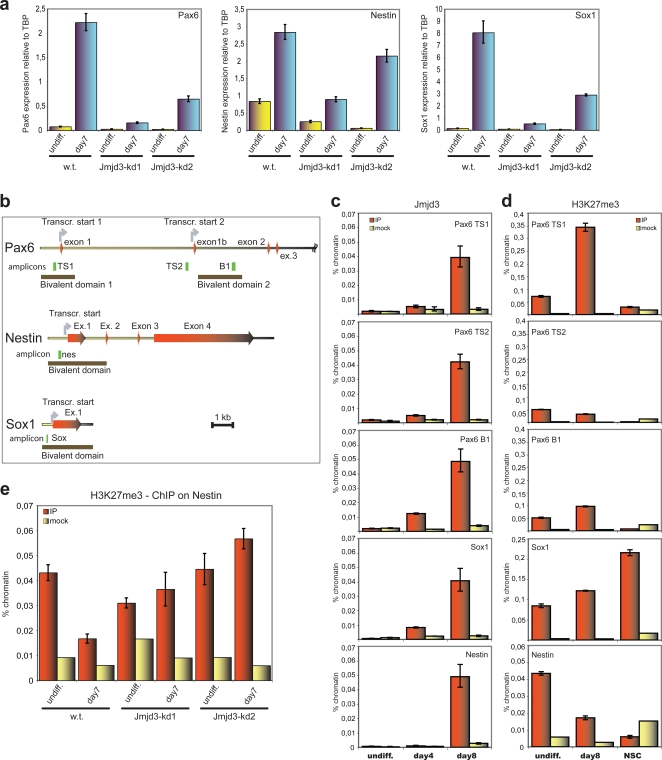
Jmjd3 regulates key effectors of neurogenesis. (a) Loss of Jmjd3 impairs upregulation of the neuronal markers Pax6, Nestin and Sox1 during differentiation. Levels of Pax6, Nestin and Sox1 were quantified by real-time RT-PCR. Bars represent the means±s.d. of triplicates normalized to TBP. W.t.: wild-type ES cells; Jmjd3-kd: Jmjd3 knock-down cells. (b) Scheme of the genomic regions analyzed with chromatin immunoprecipitation. The Pax6 (upper panel), Nestin (middle panel) and Sox1 (bottom panel) *loci* are drawn to scale, showing (partial) exon-intron structure, the transcription start site(s), the region covered by the so-called bivalent domains (see text) and the amplicons analyzed by qPCR after immunoprecipitation. (c) Chromatin immunoprecipitation showing levels of Jmjd3 occupancy of the genomic regions outlined in 3b in normal ES cells during differentiation. Levels of enrichment are shown as percentage of input chromatin. Error bars represent standard deviations of qPCR triplicates. *undiff*.: undifferentiated ES cells; *day4* and *day8* represent two time points of the differentiation protocol. (d) Chromatin immunoprecipitation showing levels of H3-K27me3 on the genomic regions outlined in 3b in normal ES cells during differentiation. Levels of enrichment are shown as percentage of input chromatin. Bars are the average +/−S.E.M. of triplicate independent samples. *Undiff.*: undifferentiated ES cells; *day8*: time point after the beginning of the differentiation protocol; *NSC*: neural stem cells (day 26). (e) Chromatin immunoprecipitation showing levels of H3-K27me3 on the Nestin promoter (outlined in 3b) in wild type (w.t.) and Jmjd3-kd (Jmjd3-kd1 and 2) cells in the undifferentiated state (undiff.) and at day 7 of monolayer differentiation. Levels of enrichment are shown as percentage of input chromatin. Bars are the average +/−S.E.M. of triplicate independent samples.

To investigate the molecular mechanism through which Jmjd3 is involved in ES cell neurulation, we analyzed by qRT-PCR the expression of key developmental regulators and markers of the ES to NS cell transition, such as Pax6, Nestin and Sox1. Pax6 is a homeodomain transcription factor that controls in the developing cortex the differentiation of the radial glia, the source of stem cells for both the neuronal and glial lineage, and whose markers profile is recapitulated in the ES-derived NS cells [17]. Nestin is a neurofilament protein specifically up-regulated during neural differentiation. Sox1 is a homeodomain protein with a key role in neural commitment, whose expression is up-regulated at day 3, peaks between days 6–8 and decreases to complete repression in NS cells. As shown in fig. 4a, Jmjd3-kd cells started off with lower levels of Pax6 and Nestin already in the undifferentiated state (Sox1 was not detected), and failed to appropriately up-regulate all the three markers when neural commitment set in, with the Jmjd3-kd clone 2 showing less severe reductions likely due to the lower knock-down efficiency and the greater residual upregulation of Jmjd3. The decreased upregulation of Pax6, Nestin and Sox1 paralleled the partial upregulation of Jmjd3 in knock-down clones at day 7 of differentiation (fig. 3a), suggesting that these genes could be direct targets of Jmjd3 exquisitely sensitive to the physiologic increase of its protein levels. Hence, we investigated whether Jmjd3 was physically bound to the promoters of these genes by chromatin immunoprecipitation (ChIP). For Pax6 we probed both mapped transcriptional start sites (TSS) (TS1 and TS2 corresponding to annotated ENSEMBL transcripts ENSMUST00000016435 and ENSMUST00000090391, see Fig. 4b) with three PCR primer sets, one located 200 bp upstream of TS1 and two located, respectively, 800 bp upstream and 1kbp downstream of TS2. All the corresponding amplicons are located within the previously defined bivalent domains. For Nestin and Sox1 we probed the regions located, respectively, 350 bp and 240 bp upstream of their TSS. We assessed Jmjd3 binding in undifferentiated ES cells (day 0) and at day 4 and 8 of neural differentiation (fig. 4c). Jmjd3 was found to be recruited to the regulatory regions of both Pax6 and Sox1 (where binding is already evident at day 4 with a substantial increase at day 8) and Nestin (where binding is detected only at day 8). Together with the differentiation impairment in Jmjd3-kd cells, these observations are consistent with the role of Jmjd3 as an activator of the neurogenic program, acting directly both on genes that orchestrate this program (like Pax6 and Sox1) and on genes that code for structural components of neurogenic precursors (like Nestin). In order to confirm the specificity of Jmjd3 involvement in the neurogenic program, we took advantage of a Sox1-GFP knock-in ES cell line which enables the isolation of GFP-positive neural precursors by FACS sorting [18]. At day 7 of monolayer differentiation we recovered about 56% of GFP positive neural precursors (Supplementary fig. S6) and confirmed that also in this highly homogeneous population of cells Jmjd3 is specifically recruited to the TSS of Pax6, Nestin and Sox1 (Supplementary fig. S7), whereas it is not recruited to the promoter of the Prolactin gene (Supplementary fig. S7).

### Jmjd3 targets show distinct patterns of H3-K27 methylation and expression

In ES cells Pax6, Nestin and Sox1 are marked by a bivalent H3-K4me3/H3-K27me3 domain [3], [10], a chromatin signature that is dynamically regulated during differentiation. Hence, the observation that Jmjd3 is recruited to their promoters prompted us to assess the dynamic profile of H3-K27 methylation at the TSS of Pax6, Nestin and Sox1 (fig. 4d) during differentiation. For all three genes our detailed analysis confirmed the observation [3], [10] that the bivalent domains of Pax6 and Nestin are resolved in NS cells (H3-K27me3 is abolished, for all the amplicons tested compared to undifferentiated cells) whereas Sox1 retained the H3-K27me3 mark.

At the Nestin TSS the progressive reduction of H3-K27me3 during differentiation (*p<0.01*) coincides with Jmjd3 occupancy suggesting a direct and causal relationship between Jmjd3 recruitment and H3-K27 demethylation. In order to prove this hypothesis, we probed the H3-K27me3 status at the Nestin TSS in Jmjd3-depleted cells. As shown in fig. 4e, whereas in wild type cells H3-K27me3 decreased by about 60-70% at day 7 of differentiation, in Jmjd3-kd clones it remained at the same level observed in undifferentiated cells (normalization for total H3 levels is shown in Supplementary fig. S8), thus providing a functional demonstration that Jmjd3 demethylates H3-K27me3 at the Nestin TSS.

For Pax6, on the other hand, we found a significant increase in H3-K27me3 both upstream of the first TSS and downstream of the second TSS (*p<0.01* in both cases), whereas basal levels of H3-K27me3 upstream of the second TSS were much lower and did not show any statistically consistent change during the first eight days of differentiation. It is noteworthy that all three regions showed a bivalent domain dynamics (both marks present in ES cells, with selective loss of H3-K27me3 in NS cells) and all three showed progressive recruitment of Jmjd3. Apparently, in the case of Pax6 initial Jmjd3 recruitment is without consequences on H3K27me3 levels and complete demethylation is observed only in NS cells, possibly to stabilize and sustain its activity. This suggests either the existence of mechanisms responsible for a late activation of its demethylase activity or the additional involvement of another H3K27me3 demethylase, a possibility we cannot formally rule out at this stage.

Finally, in the case of Sox1, whose expression is highest at days 7–8 of differentiation and is abolished in NS cells, we note a small but significant increase (*p<0.01*) in H3-K27me3 already at day 8 and a much greater increase in NS cells. This suggests that high levels of H3-K27me3 may lock in the repressed state of Sox1 in NS cells while lower levels may still allow its up-regulation at day 8, confirming that the H3-K27me3 status contributes to transcriptional output only in the context of other histone modifications and regulatory inputs.

## Discussion

Our data show that Jmjd3 is required for the differentiation of ES cells into NS cells and provide the molecular context for starting to unravel its action. H3-K27me3 is the defining mark of Polycomb-mediated epigenetic regulation. Historically, PcG genes were identified for their role in maintaining Hox silencing in *Drosophila* and mammals. This *Hox* paradigm that conflates PcG protein binding with silencing has been applied more recently to the problem of stem cell pluripotency, leading to an attractive simple model in which PcG proteins keep cells undifferentiated by silencing lineage specific genes. Genome-wide studies of PcG protein binding have revealed that indeed many developmental regulators are PcG protein targets and are repressed in ES cells, with many of them ‘held-in-check’ by H3-K4me3/H3-K27me3 bivalent chromatin domains that are later resolved in a lineage specific fashion. But these genome-wide studies have also uncovered a more complex system of regulation, in which a significant number of genes, including those associated with key stem cell pathways like Wnt, Fgf and Hedgehog, are expressed despite being PcG protein targets [5]. And from both detailed studies of PcG protein targets in *Drosophila* and genome-wide studies in mammalian cells [6], [19], [20], [21] it has become clear that H3-K27me3 at promoters is certainly compatible with transcriptional activity. Hence, these divergent observations have led to hypothesize that in stem cells PcG proteins target both genes poised for later activation and genes poised for later repression, acting as a common platform that clearly requires additional gene- and lineage-specific signals when differentiation unfolds [6], [9]. This model captures the functional essence of histone marks as molecular signals that need not only to be ‘written’ but also to be ‘read’ by the appropriate machinery.

Our characterization of Jmjd3 activity at the onset of neural commitment reflects the complexity of this regulation. It is noteworthy that current knowledge of global changes in chromatin and transcription results from a comparison between ES and NS cells, points respectively of departure and arrival for neural commitment, with similar ‘steady-state’ levels of Jmjd3. Our focus on the early stages of neural commitment, well before the stable NS cell state has been achieved, allowed us to identify Jmjd3 as a gene that is specifically up-regulated at the outset of differentiation. Recently, Jmjd3 was identified also as a target of SMRT-mediated repression in neural stem cells, and its overexpression in transformed human embryonic kidney cells (HEK cells) resulted in upregulation of neural genes, suggesting a possible role in the retinoic acid (RA)–dependent neuronal differentiation [22]. Our results expand this model and suggest that Jmjd3 fulfills a biphasic role in neurogenesis, regulating the neurogenic program both in the transition from ES to NS cells and in the further differentiation of NS cells down the neuronal lineage.

The impairment of Jmjd3-kd cells in neural commitment is reflected at the molecular level in the impaired up-regulation of key inducers and markers of neurogenesis, like Pax6, Sox1 and Nestin. And the progressive recruitment of Jmjd3 to their regulatory regions indicates that Jmjd3 directly regulates the neurogenic program of gene expression.

Interestingly, the relationship between H3-K27me3 and Jmjd3 binding at its target genes points to distinct modes of action that set the stage for further investigations. For some bivalent domain genes, exemplified here by Nestin, loss of H3-K27me3 coincides with Jmjd3 occupancy and correlates with transcriptional up-regulation, in agreement with the current paradigm of PcG-mediated silencing of lineage specific genes. Loss of Jmjd3 resulted in failure of H3-K27 demethylation, pointing to its physiologic role in demethylating this promoter. In agreement with previous reports we noted a modest increase of Nestin expression already at day 4 (data not shown), when Jmjd3 is not yet detectable at the nestin promoter. Although a trivial explanation is that the low-sensitivity of the Jmjd3 ChIP hinders the detection of low levels of Jmjd3 in the initial phases of Nestin activation, an alternative possibility is that gene induction is initiated by other mechanisms (for example recruitment of H3-K4 methyltransferases or detachment of the Polycomb repressive complex 1), and H3-K27 demethylation follows to either potentiate or maintain the induction.

For other bivalent domain genes, exemplified here by Pax6, Jmjd3 recruitment correlates with transcriptional up-regulation but apparently its H3-K27 demethylation activity sets in only later, likely to enable stable activation in NS cells. As Pax6 up-regulation is severely impaired in Jmjd3-kd cells at day 7 of differentiation, when demethylation has not yet occurred but Jmjd3 is already recruited to Pax6, it is possible that either Jmjd3 first contributes to Pax6 activation through mechanisms that are independent of its H3-K27me3 demethylase activity or that the initial effects of Jmjd3 knock-down on Pax6 expression are mediated by indirect mechanisms. The observation that the up-regulation of Pax6 (fig 4a) coincides with an increase in H3-K27me3 at its regulatory regions expands previous observations from both flies and mammals and reveal that the presence or even an increase in H3-K27me3 is compatible not simply with basal transcription but also with up-regulation, suggesting that the H3-K27 methylation status may fulfill different regulatory functions at different genes and in different cellular contexts. Finally, Jmjd3 is also recruited to developmental regulators that retain bivalent domains, like Sox1. Also in this case the presence of H3-K27me3 is compatible with a pronounced transcriptional up-regulation. As Sox1 is repressed in NS cells when its H3-K27me3 levels peak, it is possible that Jmjd3 recruitment at day 8 may prevent or mitigate an increase in H3-K27 trimethylation thereby allowing unrestrained activation mediated by other cofactors. Interestingly also other HDMs localize at promoters enriched in the methylation mark that they are competent to erase, as in the case of Jarid1a (also known as Rbp2) and Utx that occupy a subset of, respectively, H3K4me3 and H3K27me3 enriched promoters [13], [23]. Hence, our data on Jmjd3 expand these observations and further strengthen the model in which HDMs operate also, if not primarily, in the modulation rather than the simple erasure of histone lysine methylation marks.

In conclusion, our findings establish Jmjd3 as a H3-K27 demethylase required for neural commitment. The dynamics of H3-K27 demethylation, and its relationship to transcriptional activity, appear to follow distinct, gene-specific patterns and prompt further investigations into the changes of this chromatin mark at the onset of differentiation.

## Methods

### Lentivirus-mediated RNA interference

Synthetic oligonucleotides were cloned into the pSICO-R-pgkPuro lentiviral [24] and MSCV retroviral vectors [25] to express Jmjd3 and luciferase RNAi hairpins (sequences provided in the additional methods section). Production of viruses and infection of ES cells were performed according to the original publications.

### Cell culture and ES cells differentiation

E14Tg2a mouse ES cells were cultured without feeders in standard ES medium supplemented with LIF [26]. Differentiation to neural stem (NS) cells in adherent monolayer was performed as described [11].

### qPCR

qPCR was performed on ABI-Prism termocyclers using SYBR Green or Taqman chemistry. The complete list of primers is available in the additional methods section.

### Chromatin Immunoprecipitation

ChIP was performed according to standard procedures [27].

Details of all experimental procedures are included in Supplementary Information S1.

## Supporting Information

Figure S1 Specificity of the anti-Jmjd3 polyclonal antibody. Upper panel: Western blot with our anti-Jmjd3 polyclonal antibody on untransfected 293 cells (lane 1) and 293 cells overexpressing the full-length FLAG-tagged Jmjd3 protein (lane 2). Lower panel: the same samples probed with anti-vinculin as loading control.
(0.36 MB TIF)

Figure S2 In vivo H3K27me3 demethylation by Jmjd3. Full length Jmjd3 and a mutated version carrying a His_1388 to Ala mutation in the iron-binding center of the catalytic site of the JmjC domain were overexpressed in HEK-293 cells as FLAG-tagged fusion proteins. Overexpression of wild type FLAG-Jmjd3 (second lane, w.t.) results in specific loss of H3K27 trimethylation, whereas H3K27 trimethylation levels are unaffected in cells overexpressing the mutated form (third lane, mut), thus confirming that loss of H3K27 trimethylation in FLAG-Jmjd3 overexpressing cells is due to the enzymatic activity of Jmjd3. An extract of cells transfected with the empty FLAG expression vector is shown in the first lane (mock). Specificity was confirmed by immunoblotting with antibodies for specific methylated lysines.
(0.80 MB TIF)

Figure S3 In vitro demethylation by recombinant Jmjd3. The C-terminus of Jmjd3, encompassing aminoacids 1141 through 1641 and fused to a 6XHis tag, was expressed in bacteria and incubated with calf thymus histones to assess demethylation. As shown in the second lane, recombinant Jmjd3 demethylates efficiently trimethylated H3-K27, with a lesser activity on dimethylated and no activity on monomethylated H3-K27. Demethylation by Jmjd3 is dependent on iron, as shown by the reaction presented in the third lane, which was run in the absence of iron. The first lane shows the negative control reaction, in which neither recombinant Jmjd3 nor iron were added to the histone substrates. Methylation was detected by immunoblotting with antibodies specific for mono-, di- and trimethyl H3-K27. The bottom panel shows immunoblotting with anti-H3 antibody to control for the total amount of histone H3.
(0.61 MB TIF)

Figure S4 Levels of Oct4 and Nanog in undifferentiated Jmjd3-kd clones. qRT-PCR analysis of Oct4 and Nanog levels in undifferentiated wild type and Jmjd3-kd clones. Bars represent the means±s.d. of triplicates normalized to TBP.
(0.75 MB TIF)

Figure S5 Immunostaining for nestin and pan-cytokeratins of control cells expressing the luciferase RNAi hairpin. Immunostaining for nestin (left panel, second row) and pan-cytokeratins (right panel, second row) and phase-contrast images (first rows of both panels) of control cells expressing the luciferase RNAi hairpin at day 7 of monolayer differentiation.
(2.99 MB TIF)

Figure S6 FACS scan of Sox1-GFP ES cells at day 7 of monolayer differentiation. The upper panel shows the pre-sorting FACS profile of Sox1-GFP neural precursors. The background threshold was set with undifferentiated Sox1-GFP ES cells. The lower panel shows the sorted fractions (GFP+ on the left, and GFP- on the right).
(0.49 MB TIF)

Figure S7 Jmjd3 is specifically recruited to the promoters of Pax6, Nestin and Sox1 in sorted neural precursors at day 7 of monolayer differentiation. Chromatin immunoprecipitation showing levels of Jmjd3 occupancy of the genomic regions of Pax6, Nestin and Sox1 outlined in fig. 3b in the fraction of GFP+ sorted neural precursors (shown in supplementary figure S5). The Prolactin gene promoter does not show any enrichment for Jmjd3. Levels of enrichment are shown as percentage of input chromatin. Bars are the average +/−S.E.M. of triplicate independent samples.
(0.52 MB TIF)

Figure S8 Ratio of H3K27me3 to total H3 levels at the Nestin promoter on day 7 of differentiation. Chromatin immunoprecipitation showing the ratio of H3-K27me3 to total H3 levels on the Nestin promoter (outlined in 3b) in the same samples of differentiated wild type (w.t.) and Jmjd3-kd (Jmjd3-kd1 and 2) cells shown in fig. 3e. Levels of enrichment are normalized to total H3. Bars are the average +/−S.E.M. of triplicate independent samples.
(0.33 MB TIF)

Supplementary Information S1 (0.05 MB DOC)

## References

[1] Byvoet P, Shepherd GR, Hardin JM, Noland BJ (1972). The distribution and turnover of labeled methyl groups in histone fractions of cultured mammalian cells.. Arch Biochem Biophys.

[2] Azuara V, Perry P, Sauer S, Spivakov M, Jorgensen HF (2006). Chromatin signatures of pluripotent cell lines.. Nat Cell Biol.

[3] Bernstein BE, Mikkelsen TS, Xie X, Kamal M, Huebert DJ (2006). A bivalent chromatin structure marks key developmental genes in embryonic stem cells.. Cell.

[4] Saccani S, Natoli G (2002). Dynamic changes in histone H3 Lys 9 methylation occurring at tightly regulated inducible inflammatory genes.. Genes Dev.

[5] Boyer LA, Plath K, Zeitlinger J, Brambrink T, Medeiros LA (2006). Polycomb complexes repress developmental regulators in murine embryonic stem cells.. Nature.

[6] Bracken AP, Dietrich N, Pasini D, Hansen KH, Helin K (2006). Genome-wide mapping of Polycomb target genes unravels their roles in cell fate transitions.. Genes Dev.

[7] Lee TI, Jenner RG, Boyer LA, Guenther MG, Levine SS (2006). Control of developmental regulators by Polycomb in human embryonic stem cells.. Cell.

[8] Sparmann A, van Lohuizen M (2006). Polycomb silencers control cell fate, development and cancer.. Nat Rev Cancer.

[9] Ringrose L (2007). Polycomb comes of age: genome-wide profiling of target sites.. Curr Opin Cell Biol.

[10] Mikkelsen TS, Ku M, Jaffe DB, Issac B, Lieberman E (2007). Genome-wide maps of chromatin state in pluripotent and lineage-committed cells.. Nature.

[11] Conti L, Pollard SM, Gorba T, Reitano E, Toselli M (2005). Niche-independent symmetrical self-renewal of a mammalian tissue stem cell.. PLoS Biol.

[12] Klose RJ, Zhang Y (2007). Regulation of histone methylation by demethylimination and demethylation.. Nat Rev Mol Cell Biol.

[13] Agger K, Cloos PA, Christensen J, Pasini D, Rose S (2007). UTX and JMJD3 are histone H3K27 demethylases involved in HOX gene regulation and development.. Nature.

[14] De Santa F, Totaro MG, Prosperini E, Notarbartolo S, Testa G (2007). The histone H3 lysine-27 demethylase Jmjd3 links inflammation to inhibition of polycomb-mediated gene silencing.. Cell.

[15] Lan F, Bayliss PE, Rinn JL, Whetstine JR, Wang JK (2007). A histone H3 lysine 27 demethylase regulates animal posterior development.. Nature.

[16] Lee MG, Villa R, Trojer P, Norman J, Yan KP (2007). Demethylation of H3K27 regulates polycomb recruitment and H2A ubiquitination.. Science.

[17] Gotz M, Stoykova A, Gruss P (1998). Pax6 controls radial glia differentiation in the cerebral cortex.. Neuron.

[18] Ying QL, Stavridis M, Griffiths D, Li M, Smith A (2003). Conversion of embryonic stem cells into neuroectodermal precursors in adherent monoculture.. Nat Biotechnol.

[19] Papp B, Muller J (2006). Histone trimethylation and the maintenance of transcriptional ON and OFF states by trxG and PcG proteins.. Genes Dev.

[20] Ringrose L, Ehret H, Paro R (2004). Distinct contributions of histone H3 lysine 9 and 27 methylation to locus-specific stability of polycomb complexes.. Mol Cell.

[21] Roh TY, Cuddapah S, Cui K, Zhao K (2006). The genomic landscape of histone modifications in human T cells.. Proc Natl Acad Sci U S A.

[22] Jepsen K, Solum D, Zhou T, McEvilly RJ, Kim HJ (2007). SMRT-mediated repression of an H3K27 demethylase in progression from neural stem cell to neuron.. Nature.

[23] Pasini D, Hansen KH, Christensen J, Agger K, Cloos PA (2008). Coordinated regulation of transcriptional repression by the RBP2 H3K4 demethylase and Polycomb-Repressive Complex 2.. Genes Dev.

[24] Ventura A, Meissner A, Dillon CP, McManus M, Sharp PA (2004). Cre-lox-regulated conditional RNA interference from transgenes.. Proc Natl Acad Sci U S A.

[25] Dickins RA, Hemann MT, Zilfou JT, Simpson DR, Ibarra I (2005). Probing tumor phenotypes using stable and regulated synthetic microRNA precursors.. Nat Genet.

[26] Nagy AGM, Vintersten K, Behringer R (2002). Manipulating the Mouse Embryo: A Laboratory Manual. 3rd ed..

[27] Frank SR, Schroeder M, Fernandez P, Taubert S, Amati B (2001). Binding of c-Myc to chromatin mediates mitogen-induced acetylation of histone H4 and gene activation.. Genes Dev.

