# Supplementary figures and images for: The Histone H3 Lysine 27-Specific Demethylase Jmjd3 Is Required for Neural Commitment
Source: PLoS One. 2008 Aug 21;3(8):e3034. doi: 10.1371/journal.pone.0003034 (PMC2515638; doi:10.1371/journal.pone.0003034)

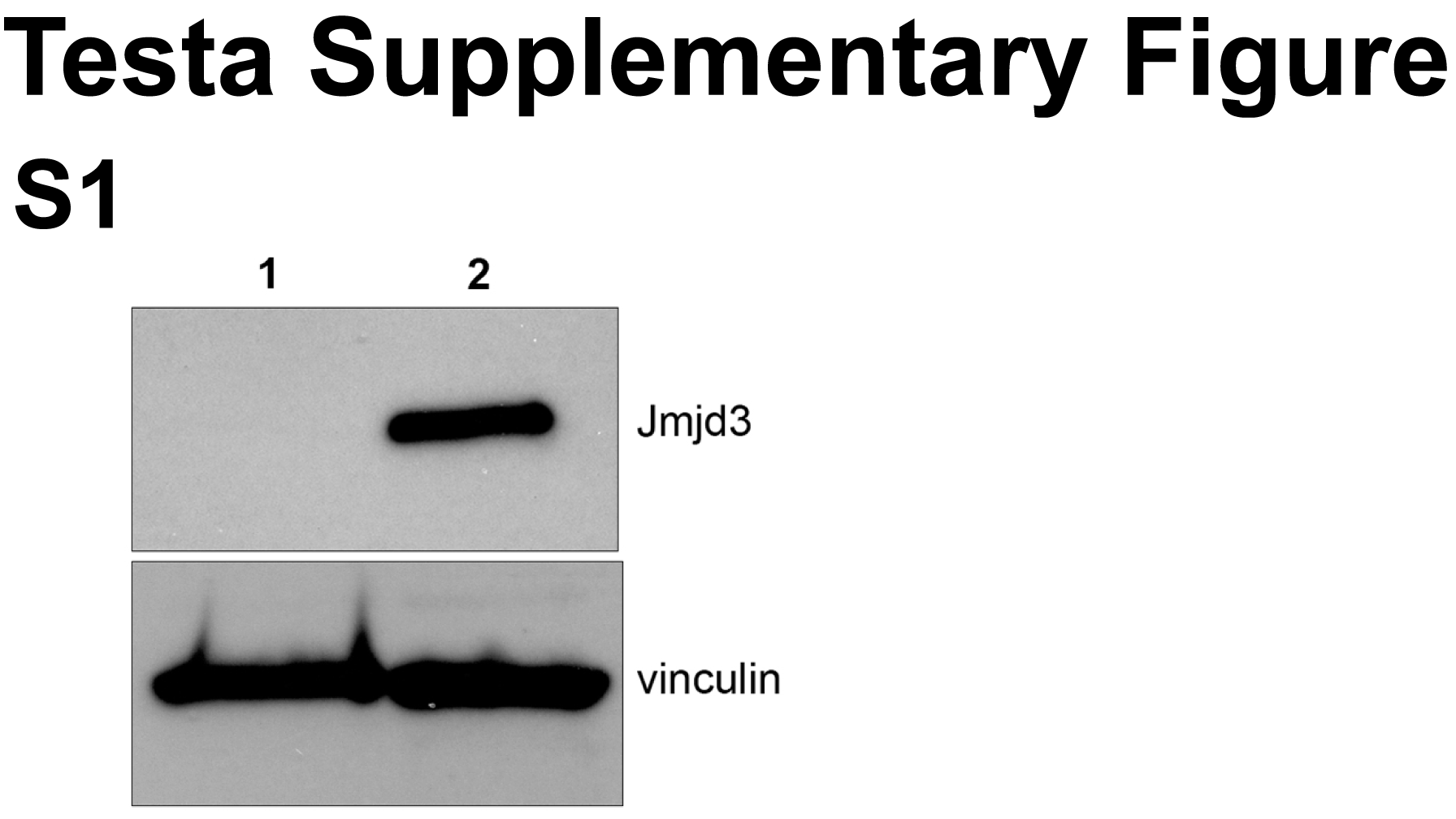

Supplement: Figure S1 — Specificity of the anti-Jmjd3 polyclonal antibody. Upper panel: Western blot with our anti-Jmjd3 polyclonal antibody on untransfected 293 cells (lane 1) and 293 cells overexpressing the full-length FLAG-tagged Jmjd3 protein (lane 2). Lower panel: the same samples probed with anti-vinculin as loading control. (0.36 MB TIF) [file pone.0003034.s001.tif]

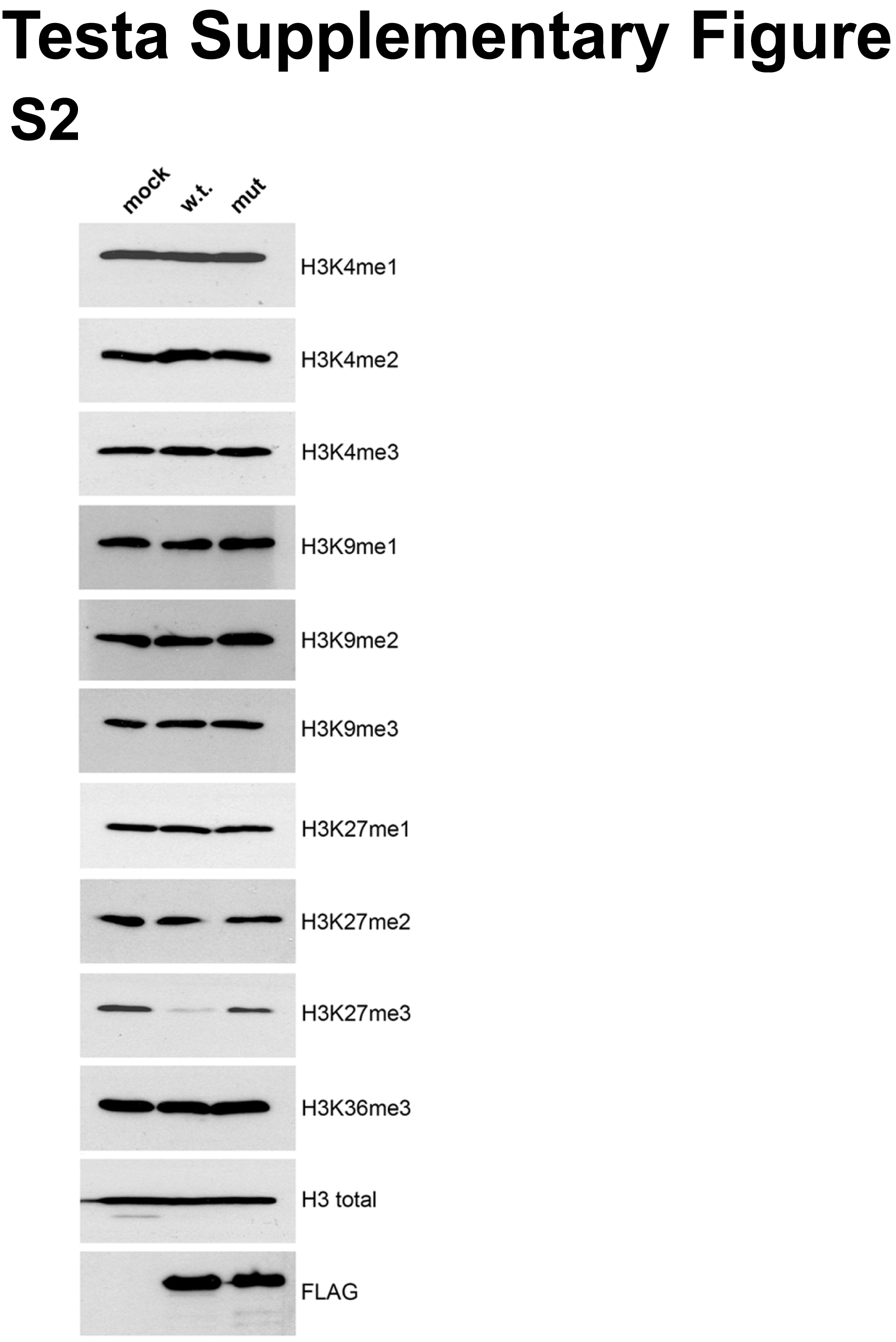

Supplement: Figure S2 — In vivo H3K27me3 demethylation by Jmjd3. Full length Jmjd3 and a mutated version carrying a His_1388 to Ala mutation in the iron-binding center of the catalytic site of the JmjC domain were overexpressed in HEK-293 cells as FLAG-tagged fusion proteins. Overexpression of wild type FLAG-Jmjd3 (second lane, w.t.) results in specific loss of H3K27 trimethylation, whereas H3K27 trimethylation levels are unaffected in cells overexpressing the mutated form (third lane, mut), thus confirming that loss of H3K27 trimethylation in FLAG-Jmjd3 overexpressing cells is due to the enzymatic activity of Jmjd3. An extract of cells transfected with the empty FLAG expression vector is shown in the first lane (mock). Specificity was confirmed by immunoblotting with antibodies for specific methylated lysines. (0.80 MB TIF) [file pone.0003034.s002.tif]

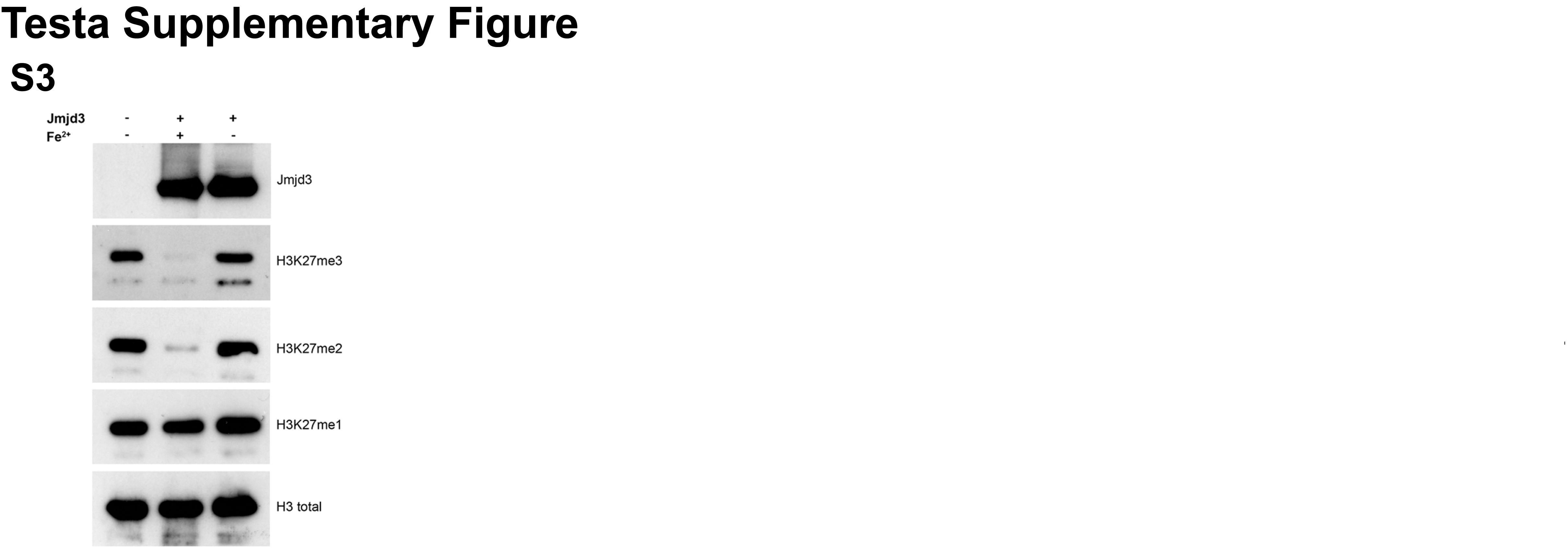

Supplement: Figure S3 — In vitro demethylation by recombinant Jmjd3. The C-terminus of Jmjd3, encompassing aminoacids 1141 through 1641 and fused to a 6XHis tag, was expressed in bacteria and incubated with calf thymus histones to assess demethylation. As shown in the second lane, recombinant Jmjd3 demethylates efficiently trimethylated H3-K27, with a lesser activity on dimethylated and no activity on monomethylated H3-K27. Demethylation by Jmjd3 is dependent on iron, as shown by the reaction presented in the third lane, which was run in the absence of iron. The first lane shows the negative control reaction, in which neither recombinant Jmjd3 nor iron were added to the histone substrates. Methylation was detected by immunoblotting with antibodies specific for mono-, di- and trimethyl H3-K27. The bottom panel shows immunoblotting with anti-H3 antibody to control for the total amount of histone H3. (0.61 MB TIF) [file pone.0003034.s003.tif]

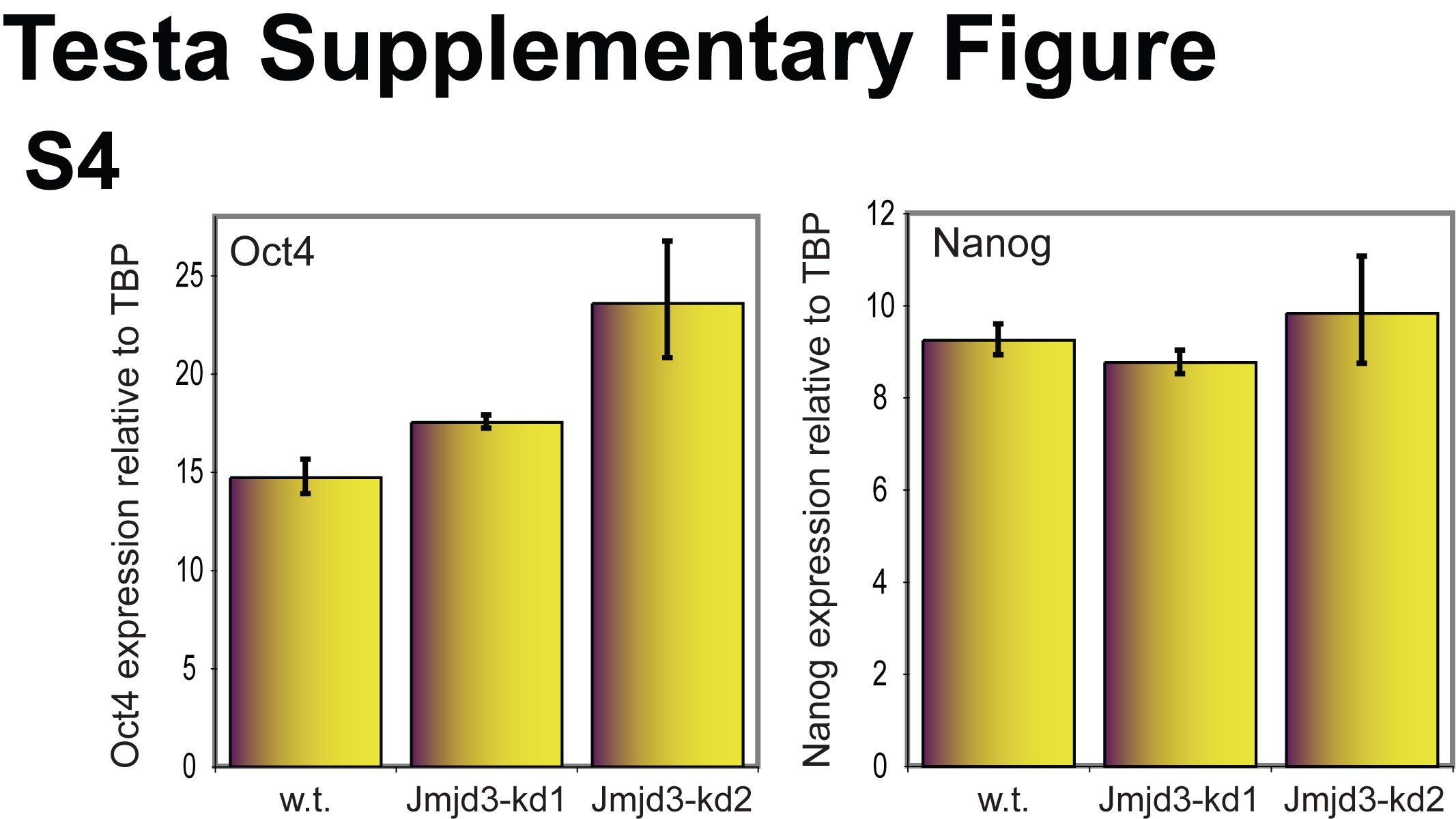

Supplement: Figure S4 — Levels of Oct4 and Nanog in undifferentiated Jmjd3-kd clones. qRT-PCR analysis of Oct4 and Nanog levels in undifferentiated wild type and Jmjd3-kd clones. Bars represent the means±s.d. of triplicates normalized to TBP. (0.75 MB TIF) [file pone.0003034.s004.tif]

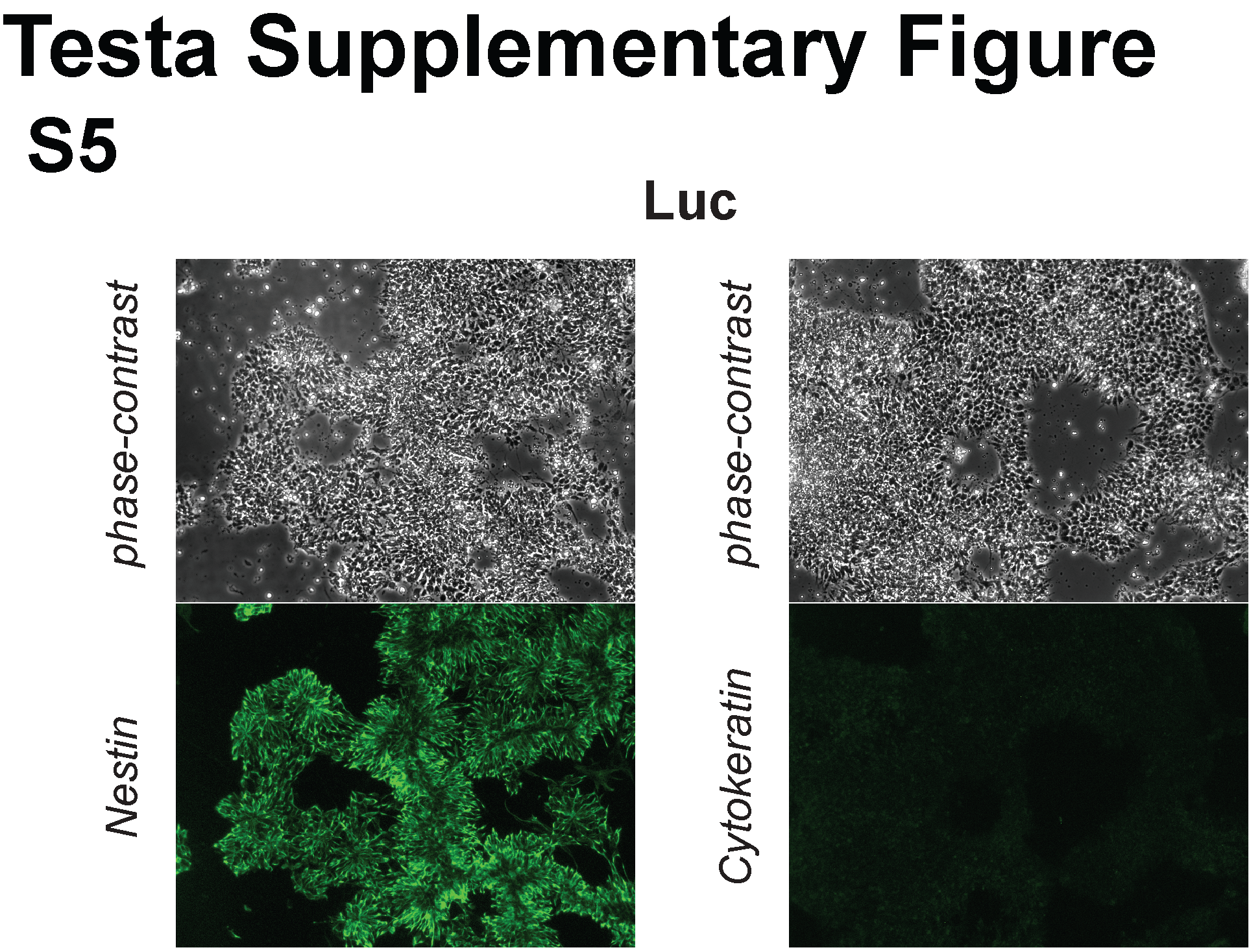

Supplement: Figure S5 — Immunostaining for nestin and pan-cytokeratins of control cells expressing the luciferase RNAi hairpin. Immunostaining for nestin (left panel, second row) and pan-cytokeratins (right panel, second row) and phase-contrast images (first rows of both panels) of control cells expressing the luciferase RNAi hairpin at day 7 of monolayer differentiation. (2.99 MB TIF) [file pone.0003034.s005.tif]

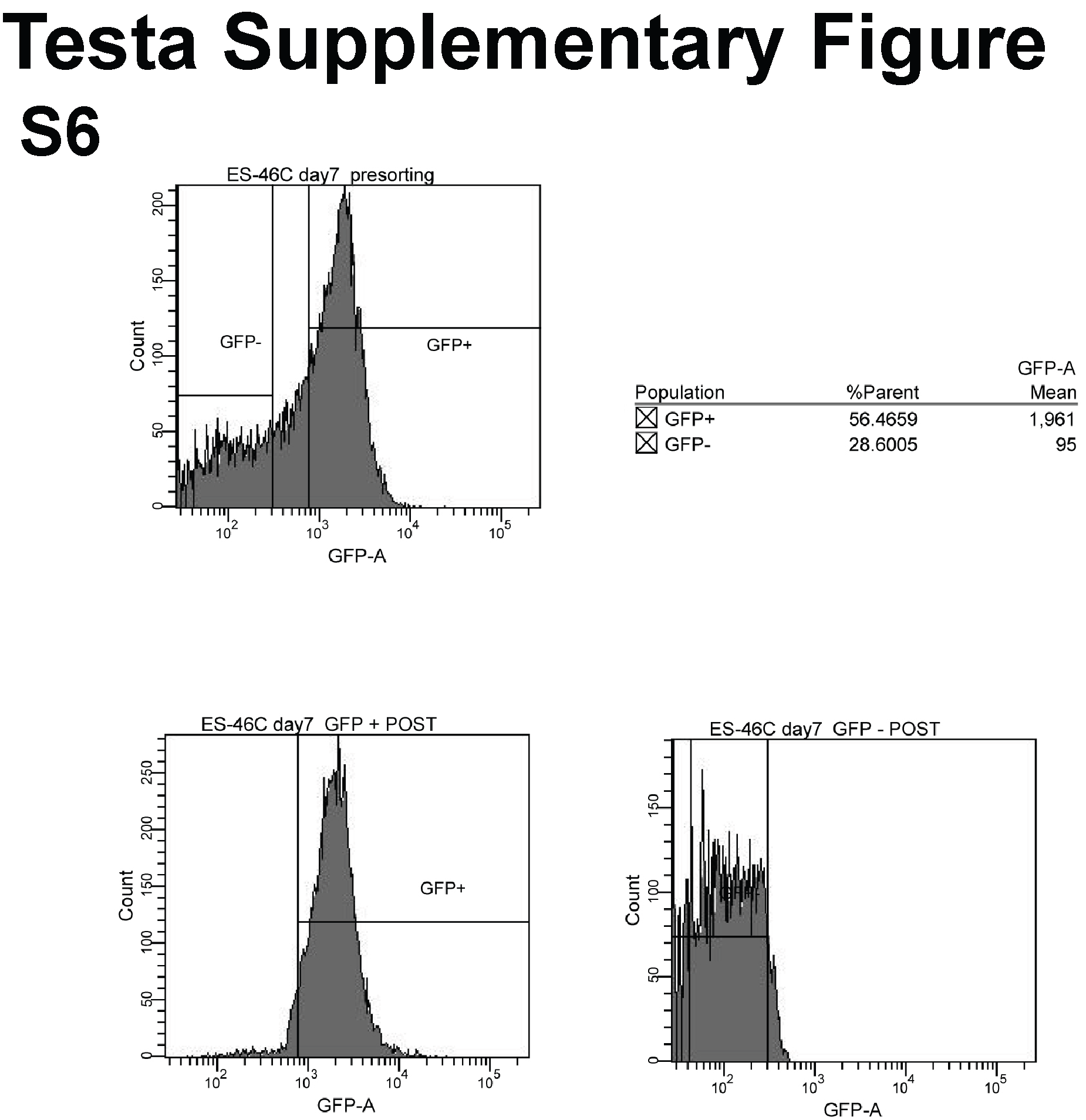

Supplement: Figure S6 — FACS scan of Sox1-GFP ES cells at day 7 of monolayer differentiation. The upper panel shows the pre-sorting FACS profile of Sox1-GFP neural precursors. The background threshold was set with undifferentiated Sox1-GFP ES cells. The lower panel shows the sorted fractions (GFP+ on the left, and GFP- on the right). (0.49 MB TIF) [file pone.0003034.s006.tif]

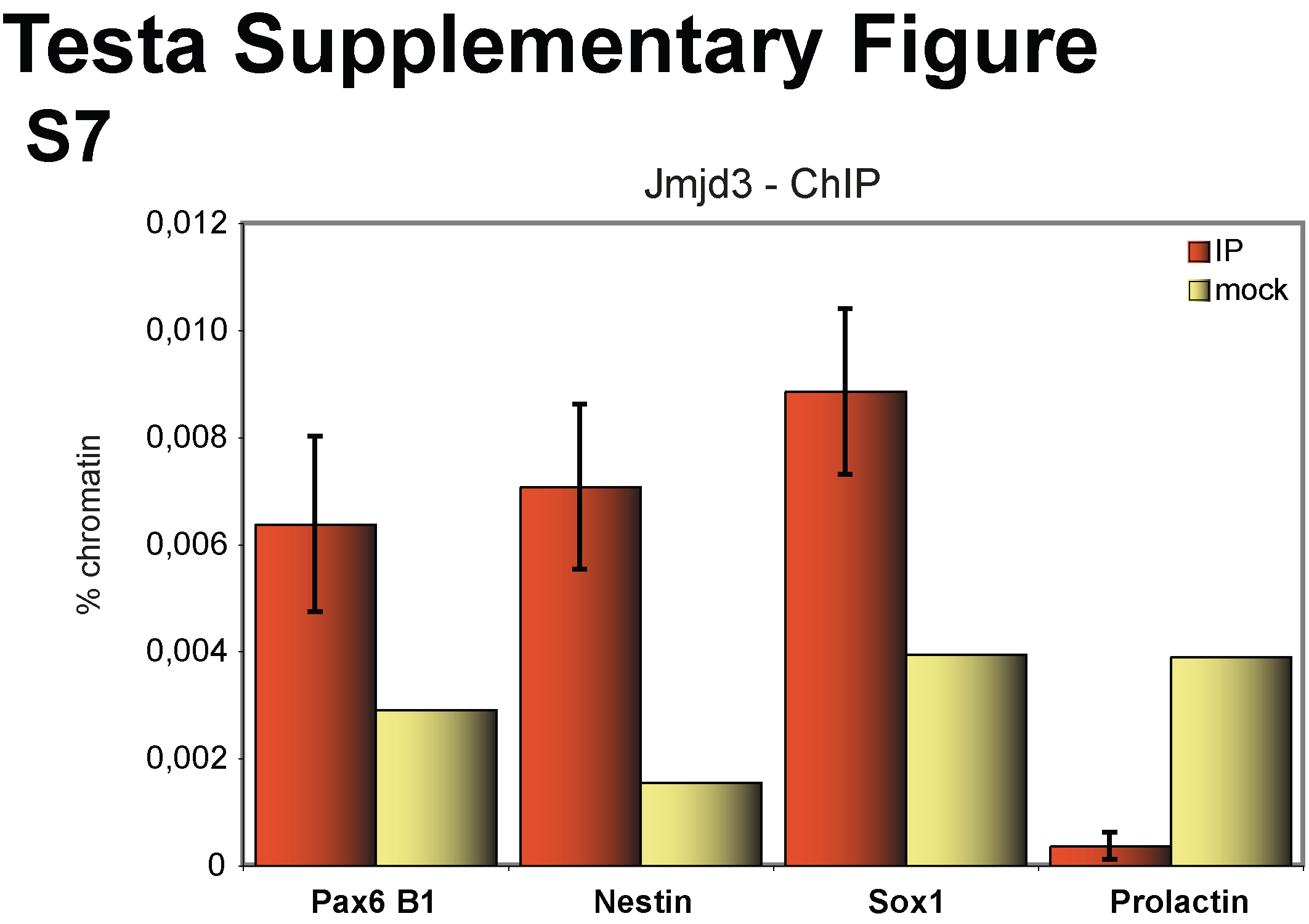

Supplement: Figure S7 — Jmjd3 is specifically recruited to the promoters of Pax6, Nestin and Sox1 in sorted neural precursors at day 7 of monolayer differentiation. Chromatin immunoprecipitation showing levels of Jmjd3 occupancy of the genomic regions of Pax6, Nestin and Sox1 outlined in fig. 3b in the fraction of GFP+ sorted neural precursors (shown in supplementary figure S5). The Prolactin gene promoter does not show any enrichment for Jmjd3. Levels of enrichment are shown as percentage of input chromatin. Bars are the average +/−S.E.M. of triplicate independent samples. (0.52 MB TIF) [file pone.0003034.s007.tif]

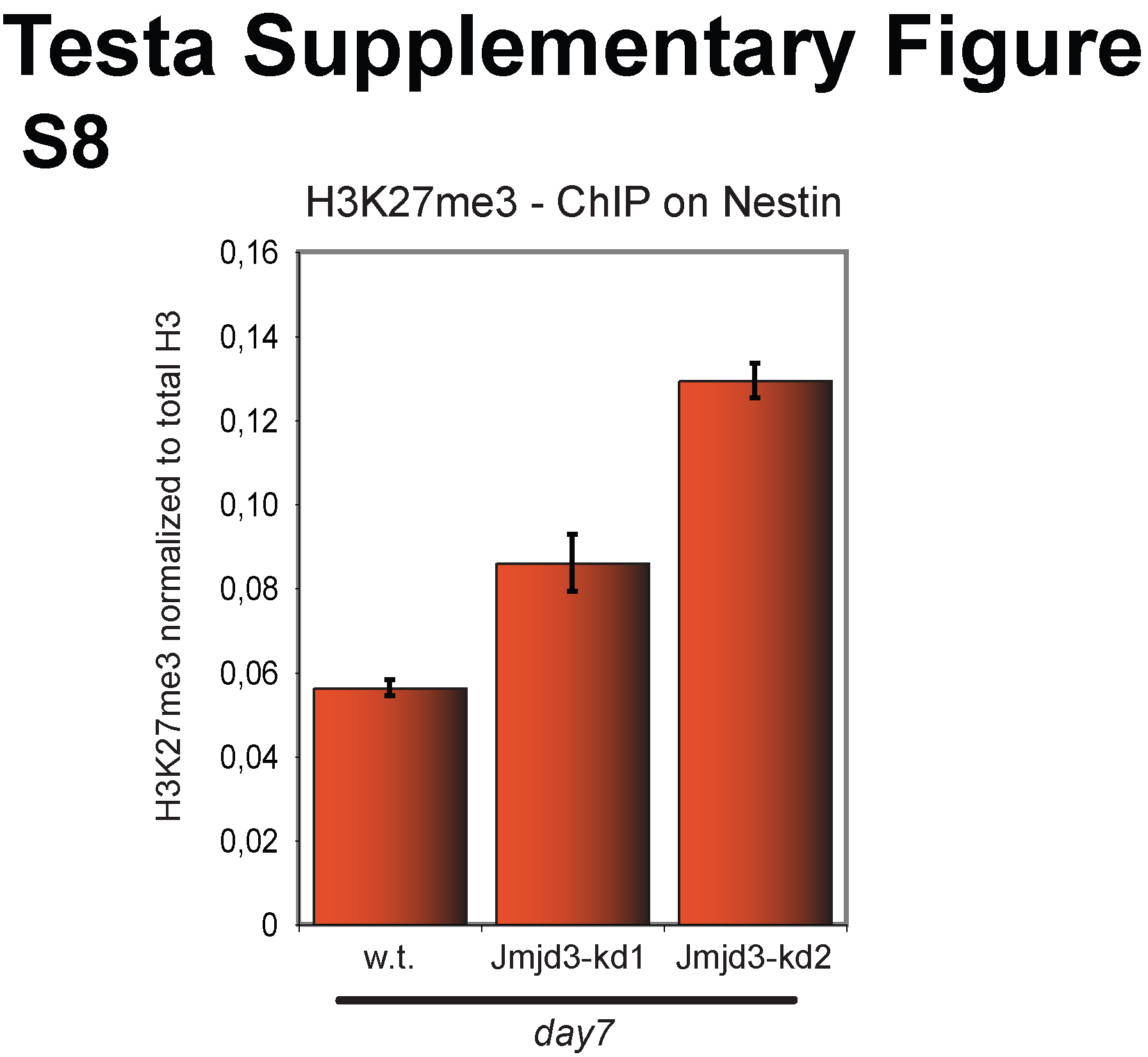

Supplement: Figure S8 — Ratio of H3K27me3 to total H3 levels at the Nestin promoter on day 7 of differentiation. Chromatin immunoprecipitation showing the ratio of H3-K27me3 to total H3 levels on the Nestin promoter (outlined in 3b) in the same samples of differentiated wild type (w.t.) and Jmjd3-kd (Jmjd3-kd1 and 2) cells shown in fig. 3e. Levels of enrichment are normalized to total H3. Bars are the average +/−S.E.M. of triplicate independent samples. (0.33 MB TIF) [file pone.0003034.s008.tif]
