# Supplementary material for: The Histone H3 Lysine 27-Specific Demethylase Jmjd3 Is Required for Neural Commitment
Source: PLoS One. 2008 Aug 21;3(8):e3034. doi: 10.1371/journal.pone.0003034 (PMC2515638; doi:10.1371/journal.pone.0003034)
Supplement: Supplementary Information S1 — (0.05 MB DOC) [file pone.0003034.s009.doc]

**Supplementary Information**

**Index:**

1: Detailed experimental procedures

2: Oligonucleotides used to generate shRNAs targeting Jmjd3

3: Antibodies used in this study

4: Real-time PCR primers for expression analysis of murine JmjC genes

**1: Detailed experimental procedures**

**Chromatin Immunoprecipitation assay**

Adherent cells were cross-linked by adding formaldehyde (final concentration of 1%) to the culture medium. After 5 min fixation the reaction was quenched by adding Tris‑Cl pH 7.6 to a final concentration of 125 mM, cells were extensively washed in PBS and harvested by scraping. Cell membranes were disrupted in L1 swelling buffer (50 mM Tris-Cl pH 8.0; 2 mM EDTA; 0,1% NP40; 10% Glycerol; protein inhibitors cocktail; about 80 μl/10^6^ cells), nuclei precipitated and resuspended in 600 μl/10^7^ cells of nuclear lysis buffer (Tris-Cl pH 8.0; 5 mM EDTA; 1% SDS; protein inhibitors cocktail). DNA shearing was performed with a Diagenode BioRuptor sonicator (High power, 4 cycles of 30 sec puls + 30 sec rest) yielding genomic DNA fragments with a size ranging between 1 kb and 200 bp, as verified by agarose gel electrophoresis of phenol-clorophorm purified probes. After pre-clearing by incubation with blocked protein A beads (50% slurry protein A-Sepharose, Amersham; 0,2 mg/mL salmon sperm DNA in TE), samples were immunoprecipitated O/N at 4°C after dilution in 9 volumes of DB (50 mM Tris-Cl pH 8.0; 5 mM EDTA; 200 mM NaCl; 0,5% NP40; for Jmjd3 and total H3 reactions) or by adjusting lysis buffer to a final concentration of 1% Triton-X100 (H3K27me3 reactions). Antibodies against H3K27me3 and total H3 were the same used for western blot. Immune complexes were recovered by addition of 20 μl/sample of blocked protein A beads for 30 min at 4°C. Beads were washed in WB (20 mM Tris-Cl pH 8.0; 2 mM EDTA; 0,1% SDS; 1% NP40; 500 mM NaCl) and in 1 x TE, and the eluted material incubated O/N at 65°C for reversion of the cross-linking. DNA was purified by the QIAquick PCR purification Kit (QIAGEN) and eluted in 100 μL of EB. 5 μL were measured in each qPCR reaction. Data were analyzed by means of ANOVA. Statistically significant differences were never observed among samples when total H3 immunoprecipitation was applied, excluding an unspecific effect of sample composition on ChIP efficiency, therefore no normalization of the Jmjd3 or H3K27me3 reactions to the total H3 was necessary. Two-way ANOVA excluded a statistically relevant difference across different experiments in the H3K27me3 immunoprecipitation, and evidenced statistically significant differences among samples when indicated in the text.

**Quantitative PCR (qPCR)**

Mouse Pax6, Nestin, TBP, Oct4, Nanog and Sox1 were measured with the TaqMan chemistry by using Applied Biosystems Inventoried Gene Expression Assays. All other real-time PCR reactions, comprising the quantification of Chromatin Immunoprecipitation, were performed using SYBR Green chemistry. Primers Px6TS1d (GGA GGA CAA TAC CAG CCA GA) and Px6TS1r (GGT TCA GCT CGG CAG ATT AG) amplify a 144 bp-long fragment mapping in the Pax6 transcription start 1 region. Primers Px6TS2d (AAG CGA ACC GTG GCT CGG) and Px6TS2r (ATT AGC GAA GCC TGA CCT CTG) amplify a 95 bp-long fragment mapping in the Pax6 transcription start 2 region, like the primer pair B1F (AGG CAG AGG GGT CTA GCT TC9 and B1R (CAA GCA AGT GGG AAG GTG AT). Primers Nes1F (TTC TTC GGG CAG TGT TTC TT) and Nes1R (GAC GGT GCA GTG TTT TGT GT) map on the Nestin promoter, while Sox1BF (CAC AGT TCA GCC CTG AGT GA) and Sox1BR (CAC AAA CCA CTT GCC AAA GA) on the Sox1 promoter. Primers PrlF (CCT TCA TTT CTG GCC AAT GT) and PrlR (GCC TGA GAG AAC CAC AGC TT) amplify a 200 bp fragment located on the transcription start site of the Prolactin gene. Conditions were optimized in respect of specificity (absence of secondary peaks in the dissociation curve and of signals in negative controls), best primer concentration and linearity of amplification over a wide range of template concentrations as described in Nolan T, Hands RE, Bustin SA (2006) *Nat. Prot.* **1**, 1559-1582. Immunoprecipitated samples were measured in triplicate including a mock control (no antibody) and the input chromatin preparation in a total reaction volume of 25 μl. Reactions were run on a Abi-Prism 7500 termocycler and data collected by the SDS software. Dissociation curve pattern confirmed specificity of reaction in each sample. Enrichment of specific promoter regions after immunoprecipitation was calculated as a percentage of the total input chromatin following the method described by Frank S.R. *et al*, 2001.

**Immunofluorescence**

Cells were fixed in 4% paraformaldehyde, washed in PBS and incubated in blocking buffer (PBS, 2% BSA, 0.1% Triton). Primary antibodies were incubated at 4°C overnight. The concentrations of the antibodies were 1:500 for anti-Nestin and 1:100 for anti-cytokeratin 5/8.

Images were acquired with an Olympus fluorescence microscope with the ScanR automated image acquisition software. Image analysis was performed with the ImageJ software.

**FACS sorting**

ES cells and neural precursors were trypsinized and sorted in FACS buffer (PBS plus 1% FBS).

***In vivo* demethylation assay**

293 cells were transfected with pcDNA3-FLAG expressing FLAG-tagged Jmjd3 and FLAG-tagged mutated version carrying an amino acid substitution in the catalytic site, along with the empty pcDNA3-FLAG vector as control. Laemmli protein extracts were immunoblotted and assessed with antibodies specific for the various methylated lysines.

***In vitro* demethylation assay**

We cloned the C-terminus of murine Jmjd3 (comprising residues 1141 through1641) into the pETM14 expression vector in frame with a 6xHis tag, expressed in bacteria and purified it from the soluble fraction. We carried out the demethylation assay incubating 10 µg of recombinant Jmjd3 and 5 µg of calf thymus histone H3 for 4 hours in a reaction buffer containing 50 mM Tris (pH 8.0), 10% glycerol, 1 mM α-ketoglutarate, 80 mM FeSO4 and 2 mM ascorbic acid. To confirm the cofactor requirements of the reaction, no FeSO_4_ was added to the assay.

**2: Oligonucleotides used to generate shRNAs targeting Jmjd3**

Jmjd3-shRNA design 1 (Jmjd3-kd1)

5’-TACACCACCATCGCTAAATATTCAAGAGATATTTAGCGATGGTGGTGTTTTTTTC-3’

5’-TCGAGAAAAAAACACCACCATCGCTAAATATCTCTTGAATATTTAGCGATGGTGGTGTA-3’

Jmjd3-shRNA design 2 (Jmjd3-kd2)

5’- TGCTGTTGACAGTGAGCGAGCACTCGATGCCTCATTCATATAGTGAAGCCACAGATGTATATGAATGAGGCATCGAGTGCCTGCCTACTGCCTCGGA-3’

Luciferase-shRNA (Luc)

5’-

TGAGCTGTTTCTGAGGAGCCTTCAAGAGAGGCTCCTCAGAAACAGCTCTTTTTTC-3’

5’-

TCGAGAAAAAAGAGCTGTTTCTGAGGAGCCTCTCTTGAAGGCTCCTCAGAAACAGCTCA-3’

**3: Antibodies used in this study**

For Western blot analysis of Jmjd3 we raised a rabbit polyclonal antibody. Mouse monoclonal anti-vinculin (SIGMA V4505), was used for normalization. Antibodies recognizing histones were from Abcam (ab1791, H3 total; ab8895 H3K4me1; ab7766 H3K4me2; ab8580 H3K4me3; ab9050 H3K36me3; ab3594 H3K79me2) or Upstate Biotechnology (#07-450 H3K9me1; #07-441 H3K9me2; #07-442 H3K9me3; #07-448 H3K27me1; #07-452 H3K27me2; #07-449 H3K27me3).

For immunostaining, we used mouse monoclonal anti-Nestin (Chemicon MAB353), rabbit plyclonal anti-Sox2 (Abcam ab15830), rabbit monoclonal anti β-TubulinIII (Covance MRB-435P), mouse monoclonal anti Gfap (Chemicon MAB3402), mouse monoclonal anti HA (Babco) and mouse monoclonal anti cytokeratin 5/8 (BD).

**4: Real-time PCR primers for expression analysis of murine JmjC genes**
